# First report of *Anopheles stephensi* from southern Ethiopia

**DOI:** 10.1186/s12936-023-04813-x

**Published:** 2023-12-08

**Authors:** Dawit Hawaria, Solomon Kibret, Daibin Zhong, Ming-Chieh Lee, Kidane Lelisa, Belayneh Bekele, Muntasha Birhanu, Mathe Mengesha, Hiwot Solomon, Delenesaw Yewhalaw, Guiyun Yan

**Affiliations:** 1 School of Environmental Health, Hawassa University, Hawassa, Ethiopia; 2 West Valley Mosquito and Vector Control District, Ontario, CA USA; 3 Program in Public Health, University of California at Irvine, Irvine, CA 92697 USA; 4 Department of Biology, Dilla University, Dilla, Ethiopia; 5 Disease Prevention Department, Sidama Regional Health Bureau, Hawassa, Ethiopia; 6 Hawassa City Administration Health Department, Hawassa, Ethiopia; 7 Sidama Regional Public Health Institute, Hawassa, Ethiopia; 8 Ministry of Health, Addis Ababa, Ethiopia; 9 Tropical and Infectious Diseases Research Centre (TIDRC), Jimma University, Jimma, Ethiopia

**Keywords:** *Anopheles stephensi*, Malaria, Mosquito breeding, Mosquito habitat, Ethiopia

## Abstract

**Background:**

*Anopheles stephensi* is an emerging exotic invasive urban malaria vector in East Africa. The World Health Organization recently announced an initiative to take concerted actions to limit this vector’s expansion by strengthening surveillance and control in invaded and potentially receptive territories in Africa. This study sought to determine the invasion of *An. stephensi* in southern Ethiopia.

**Methods:**

A targeted entomological survey, both larvae and adult, was conducted in Hawassa City, southern Ethiopia between November 2022 and February 2023. *Anopheles* larvae were reared to adults for species identification. CDC light traps and BG Pro traps were used indoors and outdoors overnight at selected houses to collect adult mosquitoes in the study area. Prokopack aspirator was employed to sample indoor resting mosquitoes in the morning. Adults of *An. stephensi* was identified using morphological keys and then confirmed by PCR.

**Results:**

Larvae of *An. stephensi* were found in 28 (16.6%) of the 169 potential mosquito breeding sites surveyed. Out of 548 adult female *Anopheles* mosquitoes reared from larvae, 234 (42.7%) were identified as *An*. *stephensi* morphologically. A total of 449 female anophelines were caught, of which 53 (12.0%) were *An. stephensi*. Other anopheline species collected in the study area included *Anopheles gambiae *sensu lato (s.l.), *Anopheles pharoensis*, *Anopheles coustani*, and *Anopheles demeilloni.*

**Conclusion:**

This study confirmed the presence of *An. stephensi* in southern Ethiopia. The presence of both larval and adult stages of this mosquito attests that this species established sympatric colonization with native vector species such as *An. gambiae* (s.l.) in southern Ethiopia. The findings warrant further investigation on the ecology, behaviour, population genetics, and role of *An. stephensi* in malaria transmission in Ethiopia.

## Background

*Anopheles stephensi* is one of several important malaria vectors of urban environments in Southeast Asia, the Middle East, and the Arabian Peninsula [1]. Since its initial discovery in Djibouti in 2012 [2], this vector species has been found in eastern Ethiopia [3, 4], Sudan [5], Somalia [1], and Kenya [6].

Its adaptation to the urban environment [7] coupled with the rapid spread of *An. stephensi* in Eastern Africa poses serious challenges for controlling and eliminating malaria in Africa’s rapidly urbanizing nations. Malaria outbreaks have been documented following its recent colonization of the Horn of Africa region [8] and the increase in malaria incidence in Djibouti was attributed to *An. stephensi* [9]. According to geospatial modelling studies, many cities in sub-Saharan Africa have favorable environmental conditions for *An. stephensi* to proliferate, putting an additional 126 million Africans at risk of contracting malaria if the spread of *An. stephensi* is not curbed [10]. Another modelling study predicted that if tailor-made *An. stephensi* interventions are not implemented, and the number of malaria cases in Ethiopia might double [11]. The geographical range of *An. stephensi* may spread into rural areas, although it is mostly an urban vector [12]. It is, therefore, crucial to describe the rural–urban transitions as a continuum rather than as distinct contexts given the ongoing urban centre expansion in rural and urban areas.

Determining the geographic range of *An. stephensi* helps design tailored vector interventions. Given the importance of *An. stephensi* to transmit urban malaria and its potential effects on public health in Africa, the World Health Organization (WHO) recently launched an initiative to take coordinated actions to limit the vector’s spread by improving *An. stephensi* surveillance and control in Africa [1]. While the spread of *An. stephensi* has recently been documented in several localities in eastern, northeastern, and central Ethiopia [3, 4], its occurrence and distribution in the area of southern Ethiopia is still unknown. Balkew et al*.* [13] surveyed larval habitat in southern Ethiopia in 2019 and could not find *An. stephensi* in the surveys. Unfortunately, the study relayed a one-time survey that lasted only for a few days. The present study aimed to explore the invasion of *An. stephensi* in Hawassa, southern Ethiopia.

## Methods

### Study sites

**Fig. 1:**
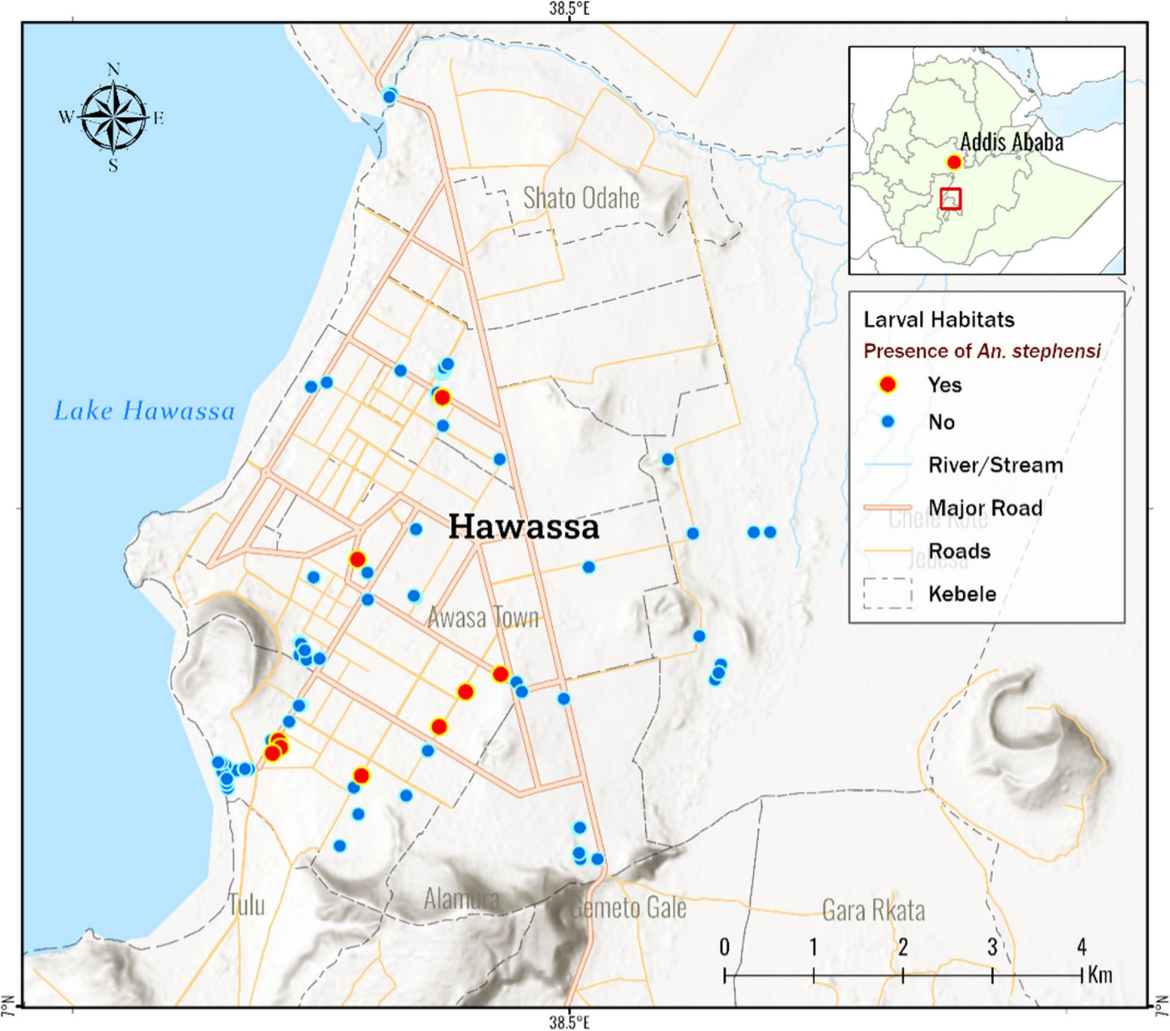
Map of the study site and *Anopheles stephensi* larval habitat distribution

Entomological survey was conducted from November 2022 to February 2023 in Hawassa City in southern Ethiopia (Fig. 1). Hawassa City is the largest city in the middle of the Great Ethiopian Rift Valley and is the capital of Sidama Regional State. It is located at an elevation of 1708 m (5604 ft) above sea level. The city’s population was 502,980 in 2022 [14]. Being situated at the shore of Lake Hawassa, the city has seen rapid growth in recent years. The city constantly sees new construction sites following its swift industrial boom in recent years. The average annual temperature and precipitation are about 21 °C and 961 mm, respectively. A shorter rainy season occurs between March and May followed by a longer wet season between July and October. Common livestock in the area include cattle, chickens, goats, sheep, donkeys, and horses. Malaria is seasonal in the area and the main malaria transmission period is between September and December [14].

### Malaria profile review

Laboratory-confirmed malaria data was obtained from the Hawassa City Health Department. The malaria morbidity data, which was sorted by age, sex, parasite species, and residence for the previous nine years was collected and analysed, and the annual transmission pattern was established.

### Mosquito larval survey and rearing

Any water collections were surveyed for mosquito larvae and pupae between November 2022 and February 2023. To increase the likelihood of discovering *An. stephensi*, target sampling was conducted. Mosquito larvae and pupae were dipped from likely larval breeding habitats, including man-made water containers, freshwater pools, lake margins, discarded tires, plastic containers, and concrete water collection tankers at construction sites. Dipping was done under larval search strategies recommended by the WHO [15]. The larvae and pupae were brought in jars to the environmental laboratory at Hawassa University, where they were placed in trays and raised to adult in preparation for morphological identification. Each enamel tray containing larvae was labeled for the habitat types from where the larvae were obtained to identify the species after adult emergence. The larvae were allowed to develop in the water drawn from the field to maintain the same aquatic environment. The pupae were sorted and transferred with pipettes from the enamel trays to beakers with modest volumes of water, then kept inside cages. A dissecting microscope was used to identify emerging adults to the species using identification key [16]. All identified specimens were preserved individually in Eppendorf tubes for further analysis.

### Adult mosquito survey

Three different types of traps were used to collect adult mosquitoes: CDC Light Traps (Model: John W. Hock CDC Light Trap 512, USA); Bioagents (BG-pro) Traps with lure; and Prokopack aspirator (John W. Hock 1418, USA). Collection with CDC Light Trap and BG-pro were made overnight from 18:00 to 06:00 h. A trap was suspended 1.5 m above the ground near a sleeping area where the people are protected by LLINs. Mosquito sampling took place both indoors and outdoors. Prokopack aspirator was employed to sample indoor resting mosquitoes in the morning from 6:30 h to 8:00 h. Animal shelters were also surveyed.

All collected adult mosquitoes were brought to the lab for species identification. Mosquitoes were killed by placing them in a refrigerator. The specimens were then sorted into culicines and *Anopheles*. Culicines were counted, recorded, and discarded. All anophelines were further sorted into species using a morphological key [16].

### Molecular identification of *An. stephensi*

A subset of the morphologically identified *An. stephensi* specimens were molecularly analyzed to confirm the species. DNA was extracted from a single leg using the Chelex method [17] with modification. Two methods were used to identify the species: (i) PCR endpoint assay using the internal transcribed spacer 2 (ITS2) locus; and (ii) sequencing portions of cytochrome c oxidase subunit 1 (*cox1*) and cytochrome B gene (*cytb*) loci. ITS2 endpoint assay was performed as previously described using the primers 5.8SB (5′-ATG CTT AAA TTT AGG GGG TAG TC-3′) and 28SC (5′-GTC TCG CGA CTG CAA CTG-3′) and the following modifications: final reagent concentrations and components were 0.5 μM for each primer; 1× DreamTaq Green Master Mix (Thermo Fisher Scientific); and water for a total reaction volume of 17 μl. PCR reaction conditions were set as denaturation at 95 °C for 3 min, 35 cycles of 94 °C for 30 s, annealing at 55 °C for 30 s, extension at 72 °C for 30 s, and a final step at 72 °C for 6 min. *Anopheles stephensi* specimens were identified by visualization of the 522-bp band with gel electrophoresis; non-*An. stephensi* specimens did not amplify and no band was present [3]. Portions of the *cox1* and *cytb* loci were also amplified for sequencing using previously detailed methods [18]. PCR products were purified and sequenced using Sanger technology by Genewiz Inc (South Plainfield, NJ). Sequences were cleaned and analysed using CodonCode (CodonCode Corporation, Centerville, MA, USA). Next, *cox1* and *cytb* sequences from *An. stephensi* were submitted as queries to the National Center for Biotechnology Information’s (NCBI) Basic Local Alignment Search Tool (BLAST) [19] against the nucleotide collection in NCBI’s GenBank. A threshold limit of 98% sequence similarity for *cox1* was used to classify sequences into species [20].

## Results

### Malaria morbidity

**Fig. 2:**
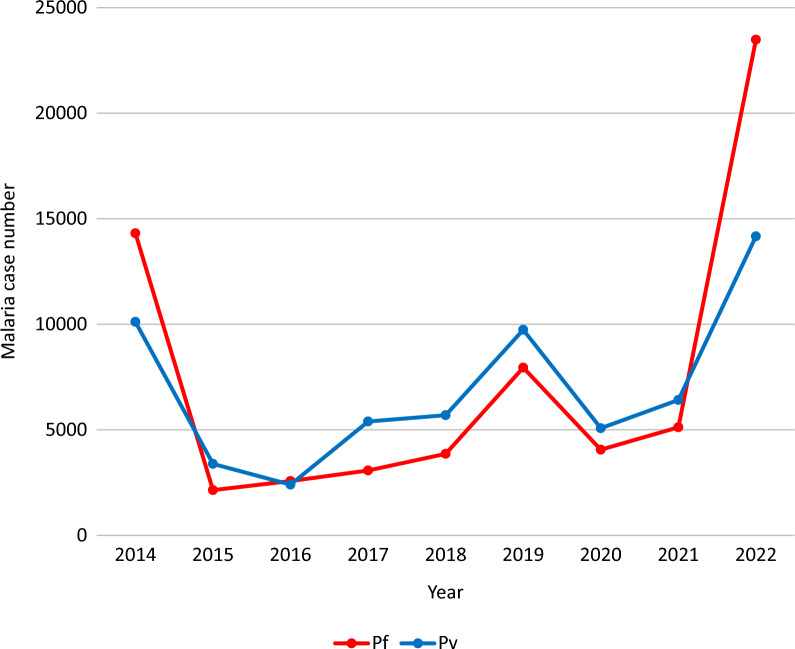
Annual malaria cases in Hawassa City, southern Ethiopia, 2023

Over the last nine years, 128,946 malaria cases were reported in the study area. *Plasmodium falciparum* and *Plasmodium vivax* were prevalent in the area and nearly had identical proportions with 66,570 (51.6%) and 62,376 (48.4%), respectively. According to the data, malaria transmission dropped for three years in a row, from 2014 to 2016. However, from 2017 to 2019, there was a slight increment. Then, after a drop in the year 2020 number of malaria cases rose steeply in 2022 (Fig. 2).

### Larval habitat distribution, positivity, and *Anopheles* species composition

*Anopheles stephensi* larvae were detected in 28 (16.6%) of the 169 mosquito breeding sites surveyed. Breeding habitats where *An. stephensi* discovered were water tanks, including plastic and metal, concrete water cisterns at construction sites, and car wash facilities (Table 1). During the survey, 2012 *Anopheles* larvae were collected and reared, and 548 (27.7%) adult females *Anopheles* emerged. Morphologically, 234 (42.7%) of 558 were identified as *An. stephensi*. Other *Anopheles* species identified include *Anopheles gambiae *sensu lato (s.l.) (n = 167; 30.5%), *Anopheles pharoensis* (n = 98; 17.9%), and *Anopheles coustani* (n = 49; 8.9%). Figure 1 depicts the distribution of the breeding habitats of *An. stephensi*.

**Table 1:** *Anopheles* mosquito larval habitat positivity and species composition by habitat types, Hawassa, southern Ethiopia, 2023

Habitat types	Surveyed habitats n (%)	Positive for any mosquito larvae n (%)	Positive for *Anopheles* larvae n (%)	Positive for *An. stephensi* n (%)	*Anopheles* spp.
Water tanks	57 (31.5)	24 (42.1)	20 (35.1)	20 (35.1)	*An. stephensi*
Concrete water cisterns at construction sites	44 (26.2)	20 (45.5)	11 (25.0)	6 (13.6)	*An. stephensi* and *An. gambiae* (s.l.)
Concrete water collection box for carwash	10 (6.0)	3 (30.0)	2 (20.0)	2 (20.0)	*An. stephensi*
Shoreline/lake edge	9 (5.4)	8 (88.9)	7 (77.7)	No	*An. gambiae* (s.l.), *An. pharoensis*, and *An. coustani*
Drainage ditch	8 (4.8)	4 (50.0)	2 (25.0)	No	*An. gambiae* (s.l.)
Discarded buckets	7 (4.2)	3 (42.8)	2 (28.6)	No	*An. gambiae* (s.l.)
Manmade pools	5(3.0)	No	No	No	
Swamps/marshes	4 (2.4)	4 (100.0)	4 (100.0)	No	*An. gambiae* (s.l.), *An. pharoensis*, and *An. coustani*
Discarded tires	4 (2.4)	2 (50.0)	No	No	
Tire tracks/road paddles	4 (2.4)	2 (50.0)	2 (50.0)	No	*An. gambiae* (s.l.)
Excavated ground for road construction	3 (1.8)	3 (100.0)	3 (100.0)	No	*An. gambiae* (s.l.)
Others	14 (8.3)	7 (50.0)	1 (7.1)	No	

*no* not observed, *spp.* species

### Adult collection’s *Anopheles* species composition

**Fig. 3:**
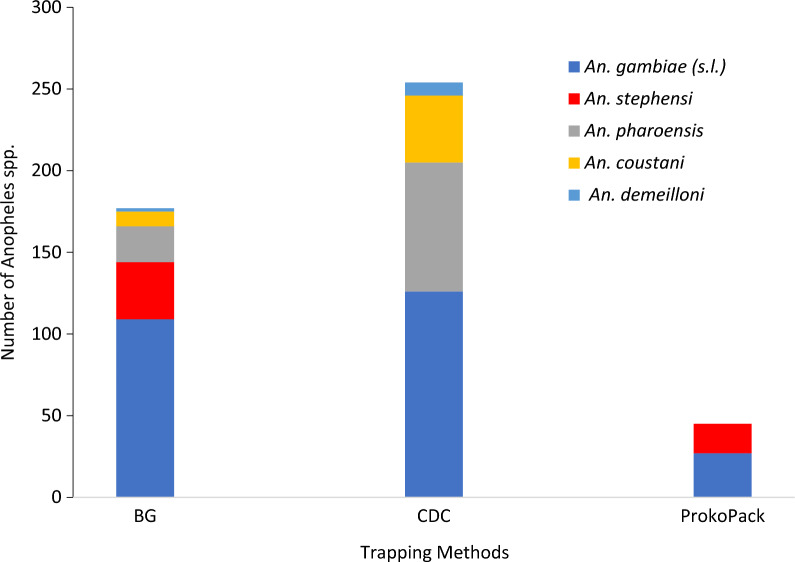
Number of *Anopheles* species caught in different trapping methods, Hawassa City, southern Ethiopia, 2023

The adult survey captured 449 female *Anopheles*, 53 (12.0%) of them were *An. stephensi*. *Anopheles stephens*i was captured by the BG Pro Trap and the ProkoPack aspirator. From 53 *An. stephensi*, 33 (62.3%) were collected indoors and 20 (37.7%) were outdoors; 38 (71.6%) of them were blood-fed. No *An. stephensi* was captured by the CDC light trap*. Anopheles gambiae* s.l., *An. pharoensis*, *An. coustani*, and *Anopheles demeilloni* were other *Anopheles* species collected during the survey. *Anopheles gambiae* s.l. was the predominant species (53.7%) followed by *An. pharoensis* (23.3%) and *An. coustani* (11.5%) (Fig. 3).

### Molecular identification of *An. stephensi*

Of the 50 morphologically identified *An. stephensi* specimens analysed, ITS2 PCR results were obtained from all of them. The morphological identification to the ITS2 PCR endpoint assay results were compared. With the PCR endpoint assay, 48 (96%) specimens were confirmed as *An. stephensi*. Only two of the 50 (4.0%) morphologically identified *An. stephensi* were not confirmed with the PCR endpoint assay. Sanger sequencing was performed for 30 confirmed and 2 unconfirmed specimens. Of the 30 *An. stephens*i, 11 were found to be cox1 haplotype 2 (GenBank accession OQ865406) and cytb haplotype 2 (GenBank accession OQ863377), while 19 were cox1 haplotype 3 (GenBank accession OQ865407) and cytb haplotype 1 (GenBank accession OQ863376). The two specimens not confirmed by ITS2 PCR were identified as *Anopheles arabiensis* by *cox1* sequencing.

## Discussion

The recent colonization of *An. stephensi* in Eastern Africa has called for additional molecular surveillance to delineate the expansion of this new species in Africa [3, 4]. To the best of our knowledge, this is the first report to confirm the presence of *An. stephensi* in southern Ethiopia. Recent increases in malaria transmission in this region coincided with the expansion of this species in Ethiopia.

Such rapid spread of *An. stephensi* to many areas of Ethiopia raises the question of whether it is truly new arrival or has been present for a while, but gone unnoticed. Scholars have forwarded several hypotheses, one of which is that *An. stephensi* was recently brought to Ethiopia from the neighbouring country Djibouti, which Ethiopia uses as a primary commodity importation route [10]. According to the recent data, the Ethiopian isolate is most closely related to an isolate from Pakistan [4]. The second hypothesis involved *An. stephensi* being present in Ethiopia for an extended period yet has gone unnoticed [20]. Given the existing gaps in molecular vector surveillance, it is necessary to carefully re-examine the trend of entomological surveillance practices that are entirely based on morphological keys for species identification. The morphological similarity of *An. stephensi* to other *Anopheles* species, such as *An. arabiensis* as documented in this study, plus the infrequent utilization and cost of molecular methods for identification, such as PCR and sequencing, could explain why the species have been overlooked [4, 12]. This study was carried out in southern Ethiopia, which is far from the location where *An. stephensi* colonization was initially recorded in the country [4], suggesting that *An. stephensi* colonization is still ongoing. Recently, Balkew et al*.* [12] conducted a larval survey in southern Ethiopia in 2019 and did not find *An. stephensi* in the surveys. Unfortunately, the study was a one-time survey that lasted only for a few days. In the present study, more intensive surveys were done. This finding underscores the critical need to expand molecular vector surveillance in all malaria-prone areas to map *An. stephensi*’s geographic distribution in the country.

Water tanks at construction sites, concrete water cisterns, and concrete water collection boxes for carwashes were habitats found to harbour *An. stephensi* in the study area. All these habitats were man-made and produced as a result of urbanization. Previous studies in several countries reported that artificial containers are a common larval habitat for *An. stephensi* [3, 4, 21, 22]. Hawassa is one of Ethiopia’s major cities, which has been rapidly expanding in recent years. Massive development projects are currently undergoing, opening the door for the proliferation of new *An. stephensi* breeding environments. Building strong collaborations across many sectors, including health, trade and industry, housing agencies, education, and municipalities, is critical in achieving successful vector control responses in such metropolitan contexts. As the World Health Organization recommends, an integrated vector control strategy should be promoted in the area [1].

Discovering *An. stephensi* in Hawassa has important public health implications. A survey done in 2019 did not find *An. stephensi* in the area [12], which was reflected in the WHO’s malaria threats map as an absence [23]. The present study however delineated its presence in the area. Coincidentally, the city has witnessed a spike in malaria cases in recent years [13]. Both *Plasmodium vivax* and *P. falciparum* are actively transmitted in the study area. Due to the well-documented vectorial competence of *An. stephensi* [7], the presence of this species might have contributed to the increase in malaria transmission in the study area. Previous studies have also indicated that malaria incidence increased following the establishment of *An. stephensi* in eastern Ethiopia and Djibouti [8, 9]. However, the present study did not look into the vectorial capacity of *An. stephensi* in malaria transmission. Future studies need to determine the human blood index and *Plasmodium* sporozoite rate of this species in Ethiopia and the region. The relative contribution of *An. stephensi* to the city’s rising malaria incidence also need to be addressed as it coexists alongside native vector species such as *An. arabiensis, An. coustani*, and *An. pharoensis*. The survey result suggests the need for further study to elucidate this invasive species’ ecology, behavior, response to insecticides, and population genetics. A better understanding of the extent of its distribution and role in malaria transmission helps develop tailor-made vector intervention strategies in urban settings.

## Conclusion

*Anopheles stephensi* coexists with native vector species in southern Ethiopia. Further research is needed to determine its relative role in malaria transmission in the region. Heath authorities need to revise the existing vector control strategies to target *Anopheles stephensi* alongside the native malaria vector species.

## Data Availability

The datasets used and analysed during the current study are available from the corresponding author upon reasonable request.
